# Spinal Hemiplegia

**Published:** 1908-02-22

**Authors:** 


### Illustrative Cases.

### SPINAL HEMIPLEGIA.

We have recently described a case in which a chondrofibroma of the odontoid process compressing the left side of the spinal cord gave rise to left-sided hemiplegia and to left-sided hemi-anæsthesia; and we then discussed the question as to which side the anæsthesia is likely to be on when there is a unilateral lesion of the cord. The books teach that a right-sided cord lesion causes paralysis on the right side, and anæsthesia on the left side, of the body, and *vice versa*. The case we have previously given appeared to show that the teaching of the books is wrong, and that a unilateral cord lesion really causes, or may cause, both paralysis and anæsthesia on the same side of the body as the lesion is in the cord.

We invited readers to record any other similar cases they might be acquainted with; and we are able to recount two more, one in this issue and another later. These two cases are less complete than the first one we gave, in that they survive, and the diagnosis is therefore unconfirmed; but the clinical points of resemblance between them and the previous case are so great that there can be little doubt that there is a unilateral cord lesion in them.

The first case is that of a bricklayer's labourer, aged 24, a man who appears to have been healthy all his life, and to have suffered no syphilitic taint. For five or six weeks before admission into hospital he suffered from pain in the back of the neck, which prevented him from throwing his head back, and greatly impeded its motion from side to side. For this he was treated as an out-patient, without improvement. On November 4, after a sudden feeling of weakness which led him to sit down for a minute or two, he found a partial loss of sensation and motility in his left arm and left leg. He dragged the leg, and both it and the arm felt colder than did those of the right side.

On his admission there was partial paralysis in the left arm and left leg. He still dragged the leg in walking. He did not admit any defect of sensibility in either leg now, although there still existed some in the left arm. Reflex actions did not appear abnormal. There was no symptom of brain affection, and no optic neuritis.

The motions of the neck were very limited, and the patient presented an appearance of great stiffness in that region. A decided fullness and thickening were felt in the region of the upper cervical vertebræ. This swelling and enlargement were most conspicuous on the left side. The relative sensibility of the two arms, the two sides of the trunk, and the two legs, was tested by points of compasses, and it was found that two points were much more readily appreciated on the right side than on the left. There was no such difference between the two sides of the face. Attention was also directed to the movements of the chest, as it was reasonable to expect that a compression of the cervical regions of the spinal cord, sufficient to cause weakness of the arm and leg, would affect these movements more or less. It was found that the vesicular murmur was much less loud all over the left lung than it was over the right; and that the movements of the ribs were less marked on the left side.

The diagnosis in this case was between cerebral and spinal hemiplegia; and in adopting the latter, the points upon which chief stress was laid were: The absence of face palsy, the existence of what seemed to be considerable thickening of the ligamentous structures of the upper cervical vertebræ, especially of the left side, the absence of general cerebral symptoms, the absence of cardiac or renal lesions, and, most important, the presence of unilateral impairment of movement in the trunk muscles.

The patient had a long sojourn in hospital, during which he was treated chiefly by iodide of potassium. This was begun on November 13. By November 28 the neck was much smaller, and the movements were less impeded. A corresponding improvement took place in the condition of the limbs, especially of the leg. The change for the better continued until December 19, when, without apparent cause, increased pain, stiffness, and swelling of the neck came on, and there was simultaneous recurrence of weakness in the left arm and left leg. The right side of the body remained perfectly normal.

A blister was now ordered to the nape of the neck, and five grains of blue pill to be taken night and morning. The iodide of potassium was omitted, and fifteen grains of mercurial ointment were rubbed in over the back twice a day. By January 3 there was a tendency to salivation, but the neck was much better; it could be moved without pain. The syrup of the iodide of iron was added a little later in drachm doses three times a day; and a seton was introduced at the back of the neck. Under this treatment slow improvement continued, and on February 21 the patient left the hospital very much better in every respect. The leg seemed quite well; the arm was still a little weak, and the compass points still detected some impairment of sensation in it; the neck was still rather stiff and awkward, but it could be moved without pain.

The nature of the lesion and its future course are subjects for speculation. The three most likely hypotheses seem to be that it was due either to tuberculous caries, to gummatous infiltration, or to neoplasm. Of these, gumma and tubercle were most favoured by those who saw him. The improvement under iodides and mercury was scarcely rapid enough or marked enough to argue strongly in favour of gumma, especially when such a diagnosis would have been in direct opposition to the patient's history. Upon the whole, there was a leaning towards the view that the lesion was tuberculous, of the nature of cervical caries; but the only definite diagnosis was that the man had spinal hemiplegia from compression of the left side of the cervical spinal cord high up.

